# An Improved Adaptive IVMD-WPT-Based Noise Reduction Algorithm on GPS Height Time Series

**DOI:** 10.3390/s21248295

**Published:** 2021-12-11

**Authors:** Huaqing Xu, Tieding Lu, Jean-Philippe Montillet, Xiaoxing He

**Affiliations:** 1 School of Surveying and Mapping Engineering, East China University of Technology, 418 Guanglan Road, Nanchang 330013, China; 201910816003@ecut.edu.cn (H.X.); tdlu@whu.edu.cn (T.L.); 2 Key Laboratory for Digital Land and Resources of Jiangxi Province, East China University of Technology, Nanchang 330013, China; 3 Physikalisch-Meteorologisches Observatorium Davos/World Radiation Center (PMOD/WRC), Dorfstrasse 33, CH-7260 Davos, Switzerland; Jean-Philippe.Montillet@pmodwrc.ch; 4 Space and Earth Geodetic Analysis Laboratory (SEGAL), University of Beira Interior, 6201-001 Covilhã, Portugal; 5 School of Civil and Surveying & Mapping Engineering, Jiangxi University of Science and Technology, No. 86 Hongqi Ave., Ganzhou 341000, China

**Keywords:** variational mode decomposition, grasshopper optimisation algorithm, improved variational mode decomposition, wavelet packet transform

## Abstract

To improve the reliability of Global Positioning System (GPS) signal extraction, the traditional variational mode decomposition (VMD) method cannot determine the number of intrinsic modal functions or the value of the penalty factor in the process of noise reduction, which leads to inadequate or over-decomposition in time series analysis and will cause problems. Therefore, in this paper, a new approach using improved variational mode decomposition and wavelet packet transform (IVMD-WPT) was proposed, which takes the energy entropy mutual information as the objective function and uses the grasshopper optimisation algorithm to optimise the objective function to adaptively determine the number of modal decompositions and the value of the penalty factor to verify the validity of the IVMD-WPT algorithm. We performed a test experiment with two groups of simulation time series and three indicators: root mean square error (RMSE), correlation coefficient (CC) and signal-to-noise ratio (SNR). These indicators were used to evaluate the noise reduction effect. The simulation results showed that IVMD-WPT was better than the traditional empirical mode decomposition and improved variational mode decomposition (IVMD) methods and that the RMSE decreased by 0.084 and 0.0715 mm; CC and SNR increased by 0.0005 and 0.0004 dB, and 862.28 and 6.17 dB, respectively. The simulation experiments verify the effectiveness of the proposed algorithm. Finally, we performed an analysis with 100 real GPS height time series from the Crustal Movement Observation Network of China (CMONOC). The results showed that the RMSE decreased by 11.4648 and 6.7322 mm, and CC and SNR increased by 0.1458 and 0.0588 dB, and 32.6773 and 26.3918 dB, respectively. In summary, the IVMD-WPT algorithm can adaptively determine the number of decomposition modal functions of VMD and the optimal combination of penalty factors; it helps to further extract effective information for noise and can perfectly retain useful information in the original time series.

## 1. Introduction

With the rapid development of space observation technology, GPS has become an important observational approach in geodesy and geodynamics [1,2]. Globally distributed International GNSS Service (IGS, see Abbreviations) reference stations have accumulated nearly twenty years of coordinate time series, which provide valuable basic data for the study of the geodynamics and global tectonics of Earth’s lithosphere and mantle [3,4,5,6,7]. Moreover, GPS observable time series not only contain geophysical signals but also unmodelled errors and other nuisance parameters, making the GPS coordinate time series present a nonlinear variation that affects the performance of the estimation of site coordinates and velocity [8,9]. The study and analysis of the Global Navigation Satellite Systems (GNSS) time series are conducive to obtaining accurate positions and velocities of stations, reasonably understanding plate tectonic movements, and establishing and maintaining dynamic earth reference frames, and they contribute to the study of relevant geodynamic processes. Therefore, in the study of GNSS signal processing, how to effectively reduce the influence of various noises in the original timing signals has always been a hot research issue in GNSS timing analysis.

In terms of GNSS time series noise reduction, Ghaderpour and Pagiatakis developed a new method of spectral analysis, namely, the least-squares wavelet analysis (LSWA), which decomposes a time series into the time–frequency domain, allowing for the detection of short-duration signatures in the series [10,11,12]. In [13], they proposed a new method, the Hilbert–Huang Transform (HHT), which compared to the wavelet and Fourier analyses, offers much better temporal and frequency resolutions. In [14], the authors applied the Kalman filter to GPS data noise reduction, and the experimental results show that the Kalman filter has a good application effect on noise reduction of triple-difference observation data, but the accuracy of the system equation directly affects the filtering effect [14]. Mosavi et al. proposed the wavelet packet transform, which can decompose the low-frequency part and better process the high-frequency part of the signal. The wavelet packet transform improves the time–frequency resolution of the signal, but it cannot improve distortion phenomena such as blurring of the signal edge [15,16,17,18]. Huang et al. improved the empirical mode decomposition (EMD) algorithm and applied it to GPS time series noise reduction. The noise reduction effect was effectively improved, but certain endpoint effects and modal mixing phenomena occurred, affecting the noise reduction effect [19,20,21]. Through the optimisation of the EMD method, ensemble empirical mode decomposition (EEMD) [22,23] and the complementary ensemble empirical mode decomposition (CEEMD) method [24,25,26,27,28] are obtained. Although EEMD and CEEMD can effectively suppress the modal aliasing phenomenon, the calculation is complicated and large. Zhang et al. [29] improved the EEMD noise identification method based on the continuous mean square error criterion and verified that the method could correctly identify the boundary point between the signal and noise.

With the rapid development of time–frequency analysis methods, Dragomiretskiy et al. proposed a new signal multiscale time–frequency analysis and processing method, variational mode decomposition (VMD) [30]. This method is based on VMD to denoise mechanical signals, and the denoising effect is better than wavelet and EMD denoising methods to varying degrees. In view of the advantages of VMD in analysing complex nonlinear, multiscale and nonstationary data, its algorithm has good antinomies performance, but the number of modal functions and penalty factors in the VMD method needs to be set in advance, and the use of inappropriate parameter combinations will result in insufficient noise reduction, so it is not adaptive [31,32,33,34,35].

Saremi et al. proposed the grasshopper optimisation algorithm (GOA) in 2017 and compared it with a variety of optimisation algorithms. The results show that GOA has outstanding advantages in the optimisation of unimodal functions, multimodal functions and composite functions [36]. Since GOA considers a given optimisation problem as a black box and does not need any gradient information of the search space, this makes it a highly suitable optimisation technique for any properly formulated optimisation problem in different fields [37]. The GOA algorithm is not affected by the nonlinear or magnitude of a problem, where usually other global optimisation techniques show early convergence, it finds the best solution more efficient with faster convergence [38], and VMD is a non-recursive approach that can adaptively derive an ensemble of band-limited intrinsic mode functions (BLIMFs) from non-stationary and nonlinear signals simultaneously [39].

To solve the above problems, we propose an improved variational modal decomposition (VMD) algorithm combining wavelet packet and energy entropy mutual information as the objective function and combined it with data experiments and analysis to verify the effectiveness and universality of the proposed method.

The rest of the paper is organised as follow. Section 2 describes the background theory of VMD and WPD. The model of the IVMD and the flowchart of IVMD-WPT Algorithm are discussed in Section 3. The validity of the proposed method was verified by simulation time series and GPS height time series from the CMONOC in Section 4. The conclusions of the experiment are summarised in Section 5.

## 2. Principles and Methods

### 2.1. Basic Principles of the VMD Method

VMD is a new signal decomposition method that decomposes the input signal of f into K modal components with centre frequency $\omega_{k}$ and reconstructs the input raw signal. Therefore, the process of VMD can be regarded as the construction and solution of the constrained variational problem described in Equation (1).

(1) $$\left\{\begin{array}{l} \underset{\left\{\mu_{k}\right\},\left\{\omega_{k}\right\}}{\min}\left\{\sum_{k=1}^{K}{\left\|\partial_{t}\left[\left(\delta \left(t\right)+\frac{j}{\pi t}\right)\times \mu_{k}(t)\right]e^{-j\omega_{k}t}\right\|}_{2}^{2}\right\}\\ s.t.\sum_{k}\mu_{k}=f \end{array}\right.$$

where $\mu_{k}(t)$ is the intrinsic modal function, $\omega_{k}$ is the centre frequency of the modal function, $\delta \left(t\right)$ is the impulse function and $e^{-j\omega_{k}t}$ is the estimated centre frequency of each analytic signal.

Here, a quadratic penalty factor α and Lagrange operator λ(t) are used to render the variational problem unconstrained. α can ensure the accuracy of reconstruction in the presence of Gaussian noise, and λ(t) can ensure the tightness of constraint conditions. Therefore, the extended Lagrange expression is:

(2) $$\begin{array}{cc} L\left(\mu_{k}(t),\omega_{k},\lambda (t)\right) & =\alpha \sum_{k=1}^{K}{\left\|\partial_{t}\left[\left(\delta \left(t\right)+\frac{j}{\pi t}\right)\times \mu_{k}(t)\right]e^{-j\omega_{k}t}\right\|}_{2}^{2}\\ & {+\left\|f(t)-\sum_{k=1}^{K}\mu_{k}(t)\right\|}_{2}^{2}+\langle \lambda (t),f(t)-\sum_{k=1}^{K}\mu_{k}(t)\rangle \end{array}$$

Equation (2) is solved by using the alternating direction method of multipliers (ADMM) and $\mu_{k}{}^{n+1}(t)$, $\omega_{k}{}^{n+1}$ and $\lambda^{n+1}(t)$ are updated to find the optimal solution of the original variational problem in Equation (2). Equations (3) and (4) give the iterative formulae of each modal function $\mu_{k}(t)$ and corresponding central frequency $\omega_{k}$, respectively.

(3) $$\mu_{k}{}^{n+1}(\omega )=\frac{\hat{f}(\omega )-\sum_{i\neq k}{\hat{\mu }}_{i}(\omega )+\frac{\hat{\lambda }(\omega )}{2}}{1+2\alpha {\left(\omega -\omega_{k}\right)}^{2}}$$

(4) $$\omega_{k}^{n+1}=\frac{\int_{0}^{\infty }\omega {\left|{\hat{\mu }}_{k}(\omega )\right|}^{2}d\omega }{\int_{0}^{\infty }{\left|{\hat{\mu }}_{k}(\omega )\right|}^{2}d\omega }$$

where $\hat{f}(\omega )$, ${\hat{\mu }}_{k}(\omega )$, ${\hat{\mu }}_{k}{}^{n+1}(\omega )$ and $\hat{\lambda }(\omega )$ represent the Fourier transform of $\hat{f}(t)$, $\mu_{k}(t)$, $\mu_{k}^{n+1}(t)$, λ(t) and n is the number of iterations. Equation (5) is the iterative formula of the Lagrange operator.

(5) $${\hat{\lambda }}^{n+1}(\omega )={\hat{\lambda }}^{n}(\omega )+\tau \left[\hat{f}(\omega )-\sum_{k=1}^{K}{\hat{\mu }}_{k}^{n+1}(\omega )\right]$$

where τ is the iteration step, and Equation (6) is the convergence condition, ε is the convergence tolerance.

(6) $$\sum_{k=1}^{K}\frac{{\left\|{\hat{\mu }}_{k}^{n+1}-{\hat{\mu }}_{k}^{n}\right\|}_{2}^{2}}{{\left\|{\hat{\mu }}_{k}^{n}\right\|}_{2}^{2}}<\varepsilon$$

### 2.2. Grasshopper Optimisation Algorithm

The grasshopper optimisation algorithm (GOA) is a nature-inspired algorithm with high search efficiency and fast convergence. It simulates the predation behaviour of natural grasshopper swarms. The process of searching for food sources can be divided into two steps: exploration and development. In the exploration process, the long distance of the swarming is conducive to the global search, while in the development process, it is local. The behaviour is mathematically modelled as follows:

(7) $$X_{i}=S_{i}+G_{i}+A_{i}$$

where $X_{i}$ is the location of the *i*th grasshopper; $S_{i}$ is the interaction factor between the *i*th grasshopper and other grasshoppers; $G_{i}$ is the force of gravity on the first grasshopper; $A_{i}$ expresses wind advection. The calculation formula of $S_{i}$ is as follows:

(8) $$S_{i}=\sum_{j=1,j\neq i}^{N}s(\left|X_{j}-X_{i}\right|)\cdot d_{ij}$$

where N is the population size; $\left|X_{j}-X_{i}\right|$ is the distance between the *i*th and *j*th grasshoppers; $d_{ij}=\left(X_{j}-X_{i}\right)/\left|X_{j}-X_{i}\right|$ represents a unit vector from the *i*th to the *j*th grasshopper; s is the social forces between grasshoppers as shown below:

(9) $$s(r)=f\cdot e^{\frac{-r}{l}}-e^{-r}$$

where f is the attraction intensity between grasshoppers and l is the ratio of the attraction length.

$G_{i}$ and $A_{i}$ can be calculated by Equations (10) and (11).

(10) $$G_{i}=-ge_{g}$$

(11) $$A_{i}=ue_{\omega }$$

where g is the gravity constant; $e_{g}$ is the unit vector pointing to the centre of the earth; u is the wind force constant; $e_{\omega }$ is the unit vector of wind direction, so the $X_{i}$ expansion is as follows:

(12) $$X_{i}=\sum_{j=1,j\neq i}^{N}s(\left|X_{j}-X_{i}\right|)\frac{\left(X_{j}-X_{i}\right)}{d_{ij}}-ge_{g}+ue_{\omega }$$

In solving practical problems, gravity is usually not taken into account, the wind direction is always set to the optimisation target of $T_{d}$ and the parameter coordination global and local search process is introduced. Then, the position formula is as follows:

(13) $$X_{i}{}^{d}=c\left(\sum_{j=1,j\neq i}^{N}c\cdot \frac{U_{d}-L_{d}}{2}\cdot s(\left|X_{j}-X_{i}\right|)\cdot \frac{\left(X_{j}-X_{i}\right)}{d_{ij}}\right)+T_{d}$$

where $U_{d}$ and $L_{d}$ are the upper and lower limits of the function in dimensional space; $T_{d}$ is the optimal solution of the current grasshopper position; c is the attenuation coefficient as shown below:

(14) $$c=c_{\max}-l\cdot \frac{c_{\max}-c_{\min}}{M}$$

where $c_{\max}$ and $c_{\min}$ are the maximum and minimum values; l is the current iteration number; M is the maximum number of iterations.

### 2.3. Principle of the Wavelet Packet Algorithm

Wavelet decomposition decomposes the original time series into high frequency and low frequency through a set of high-pass and low-pass filters and then decomposes the low frequency part. By wavelet packet decomposition, the high-frequency part not involved in wavelet decomposition is further decomposed, and then the optimal wavelet basis function is selected. The time–frequency analysis effect is better than that of the wavelet function. The specific steps are as follows:

Step 1: Define φ(x) and ψ(x) as the orthogonal scaling function and its corresponding wavelet function. Set h(k) as the low-pass filter coefficient and g(k) as the high-pass filter coefficient.

(15) $$\left\{\begin{array}{l} \varphi (x)=\sqrt{2}\sum_{k\in z}h(k)\varphi (2x-k)\\ \psi (x)=\sqrt{2}\sum_{k\in z}g(k)\varphi (2x-k) \end{array}\right.$$

Let $\mu_{0}=\varphi (x)$ and $\mu_{1}=\psi (x)$ then:

(16) $$\left\{\begin{array}{l} \mu_{2n}(x)=\sqrt{2}\sum_{k\in z}h(k)\mu_{n}(2x-k)\\ \mu_{2n+1}(x)=\sqrt{2}\sum_{k\in z}g(k)\mu_{n}(2x-k) \end{array}\right.$$

Step 2: Assume subspace $U_{j}^{n}$ as the closure space of function $\mu_{n}(x)$ and subspace $U_{j}^{2n}$ as the closure space of function $\mu_{2n}(x)$ and $g_{j}^{n}\in U_{j}^{u}$. $C_{l}^{j,n}$ is the coefficient of φ(x) in the subspace, so $g_{j}^{n}(x)$ can be expressed as:

(17) $$C_{l}^{j,n}=\int_{-\infty }^{+\infty }\varphi (x)2^{j/2}\mu_{n}(2^{j}t-l)dt$$

(18) $$g_{j}^{n}=\sum_{l}C_{l}^{j,n}\mu_{n}(2^{j}x-1)$$

The wavelet packet decomposition algorithm can be obtained as:

(19) $$\left\{\begin{array}{l} C_{l}^{j,n}=\sum_{k}h(k-2l)C_{k}^{j+1,n}\\ C_{l}^{j,2n+1}=\sum_{k}g(k-2l)C_{k}^{j+1,n} \end{array}\right.$$

Step 3: Decompose the wavelet packet, perform the inverse operation and obtain the wavelet packet reconstruction expression as follows:

(20) $$C_{l}^{j+1,n}=\sum_{k}\left[h(l-2k)C_{l}^{j,2n}+g(l-2k)C_{l}^{j,2n+1}\right]$$

## 3. IVMD-WPT Algorithm

As discussed in the previous section, the key to VMD performing feature extraction on time series data is the determination of the decomposition modal number K and penalty factor α. If K is less than the number of useful components in the processed signal, it will cause insufficient data decomposition; conversely, it will cause an over-decomposition phenomenon. An improper value of the penalty factor α may lead to centre frequency overlap of the modal function; therefore, appropriate parameters must be selected.

### 3.1. Improved VMD Method

Since GPS time series data are polluted by stationary and nonstationary noise and a single indicator cannot be used to obtain signal features, the mixing of two or more single indicators provides stronger robustness [40,41]. Thus, to determine VMD parameters, the energy entropy mutual information (EEMI) index composed of energy entropy and mutual information is adopted in this paper. The sum of the EEMI of the previous two modal functions is taken as the objective function, and the VMD parameters are optimised by the GOA algorithm as shown in Equation (21).

(21) $$\left\{\begin{array}{c} fitness=\underset{\gamma =(K,\alpha )}{\min}\left\{-\sum_{i=1}^{2}EEMI\right\}\\ K\in \left[2,8\right]\\ \alpha \in [1000,10000] \end{array}\right.$$

where fitness is the objective function, γ=(K,α) is the value range of the decomposition modal number K and penalty factor α and EEMI=EE*MI, EE and MI are the energy entropy and mutual information, respectively, which can be calculated by:

(22) $$\left\{\begin{array}{c} E_{i}=\int_{-\infty }^{+\infty }{\left|imf_{i}(t)\right|}^{2}dt_{i},i=1,2,\ldots ,K\\ \sigma_{i}=E_{i}/E\\ EE=-\sum_{i=1}^{N}\left(\sigma_{i}\right)In(\sigma_{i}) \end{array}\right.$$

(23) $$MI(X;Y)=\sum_{y\in Y}\sum_{x\in X}p(x,y)\log\frac{p(x,y)}{p(x)p(y)}$$

where $imf_{i}(t)\;\left(i=1,\cdots ,K\right)$ are the modes of different frequency bands, $E_{i}=\left\{E_{1},E_{2},\ldots ,E_{K}\right\}$ is the energy distribution of the vibration signals in the frequency domain, $E=E_{1}+\cdots +E_{K}$, p(x) and p(y) are the edge probability distribution functions of X and Y, respectively, and p(x,y) denotes the joint probability distribution function of X and Y.

### 3.2. IVMD-WPT

**Figure 1:**
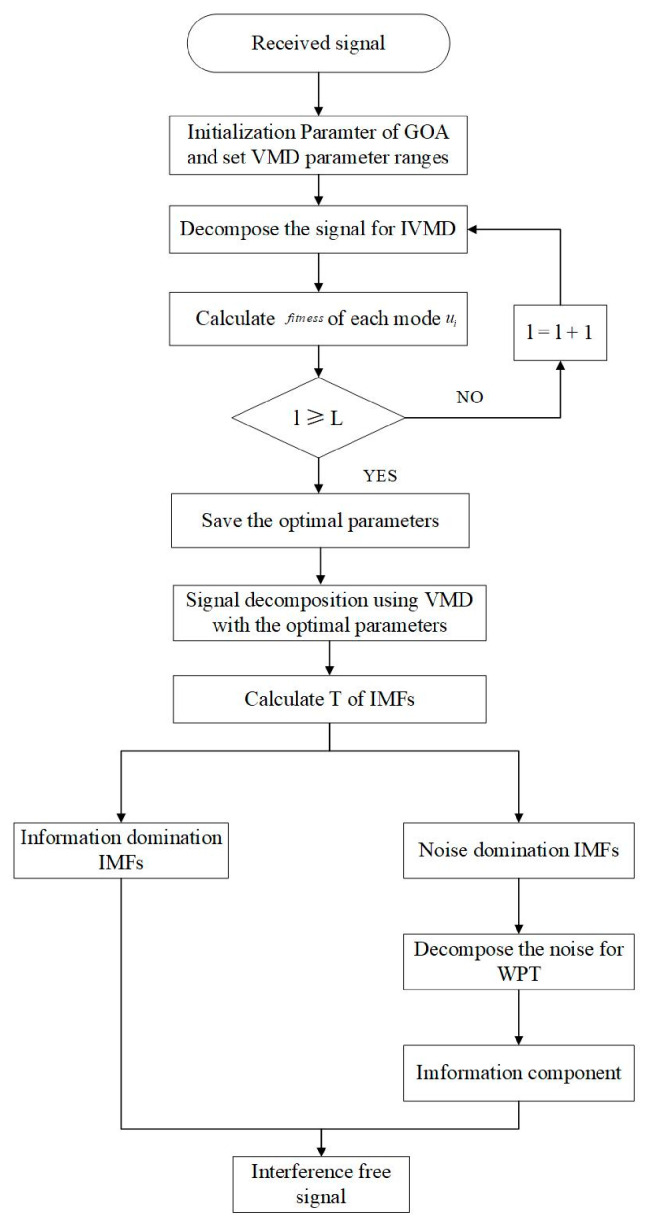
IVMD-WPT algorithm flowchart.

Aiming at the problem that the VMD method cannot determine the number of modal functions K and the penalty factor α and that the removed noise contains any valid information, this paper adopted the energy entropy mutual information (EEMI) index as the parameter adaptive to VMD and wavelet packet of the objective function. The specific steps of the combined GNSS coordinate time series noise reduction algorithm are as follows, and the flowchart is depicted in Figure 1.

Step 1: The parameter range of the VMD algorithm was set and the parameters of the GOA algorithm were initialised in [34,35] the modal component number $K\in \left[2,8\right]$, $K\in \left[2,10\right]$ and penalty factor $\alpha \in \left[1000,10000\right]$; however, in [40,41], K takes an integer in the interval of $\left[2,8\right]$, and this paper focused on VMD applying to the GPS; thus, the authors considered that the range of K and α was $\left[2,8\right]$, $\left[1000,10000\right]$, and the population number of the GOA algorithm N=30, and the maximum cycle number L=10 [34,35].

Step 2: The method described in Section 3.1 was used to select the optimal parameters of the VMD method, and the VMD method with the optimal parameters was used to decompose the GNSS coordinate time series.

Step 3: The composite evaluation index T [42] was used to form the reconstructed time series by summing each modal component successively, and the composite evaluation index T value of each reconstructed time series was calculated. When the T value was the smallest, the corresponding reconstructed time series was a denoising time series, and the remaining IMF components were regarded as high-frequency noise.

Step 4: The decomposed noise was further denoised by wavelet packet transform (WPT). Finally, the effective signal component after denoising by wavelet packet transform and the denoising time series obtained in Step 3 were reconstructed into the final denoising time series.

To effectively evaluate the effectiveness of the text combination method, the signal-to-noise ratio, root mean square error (RMSE) and correlation coefficient (R) were selected as the evaluation indices for noise reduction, and the calculation formulae are shown as follows:

(24) $$R_{sn}=10\times \log\left(\frac{\sum_{n=0}^{N-1}S_{n}^{2}}{{\sum_{n=0}^{N-1}\left(S_{n}-{\bar{S}}_{n}\right)}^{2}}\right)$$

(25) $$RMSE=\sqrt{\frac{1}{n}{\sum_{i=1}^{n}\left(x_{i}-\hat{x_{i}}\right)}^{2}}$$

(26) $$r_{i}=\frac{Cov\left[x_{i},y_{i}\right]}{\sqrt{Var\left[X\right]Var\left[Y\right]}},i=1,2,\ldots ,n$$

where n and N denote the number of sampling points in the sequence; $x_{i}$, $S_{n}$ and X denote the sequence of the signal; $\hat{x_{i}}$, ${\bar{S}}_{n}$ and Y denote the original sequence.

## 4. Experiment Analysis and Discussion

In this section, two groups of simulation data were used to verify the effectiveness of the IVMD-WPT method for denoising common periodic signals and time series signals, and GNSS measured elevation coordinate time series data were used to verify the universality of the IVMD-WPT denoising effect.

### 4.1. Simulation Experiment A

**Figure 2:**
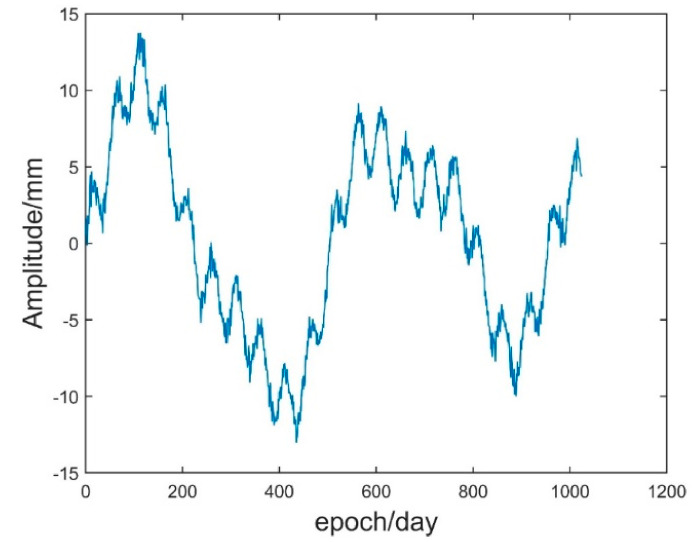
Simulation of the original time series.

**Figure 3:**
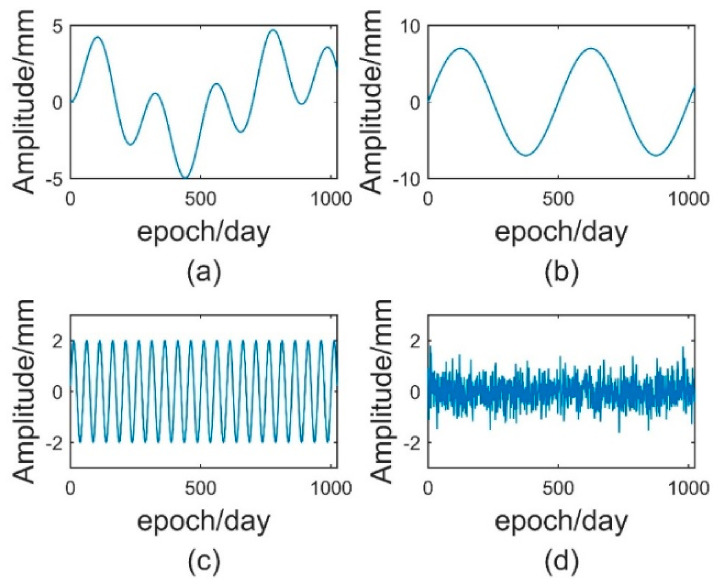
Waveform diagram of each component of the analogue signal: (**a**) waveform diagram of y1, (**b**) waveform diagram of y2, (**c**) waveform diagram of y3, (**d**) waveform diagram of noise.

Simulation GNSS elevation time series 1 (Sim_1) consists of a trend term, seasonal term (periodic term) and noise term. First, Sim_1 TS containing three constant amplitude period terms and Gaussian white noise was generated by Equation (27) in which the sampling frequency was 1 Hz, the sampling number was 1024 and the signal-to-noise ratio was 6 dB. Figure 2 and Figure 3 are the simulated original time series diagram and simulated component waveform diagram, respectively.

(27) $$\left\{\begin{array}{c} y_{1}=5\sin\left(\frac{2\pi t_{i}}{600}\right)\sin\left(\frac{2\pi t_{i}}{350}\right)\\ y_{2}=7\sin\left(\frac{2\pi t_{i}}{500}\right)\\ y_{3}=2\sin\left(\frac{2\pi t_{i}}{50}\right)\\ \varepsilon =noise\\ f=y_{1}+y_{2}+y_{3}+\varepsilon \end{array}\right.$$

**Figure 4:**
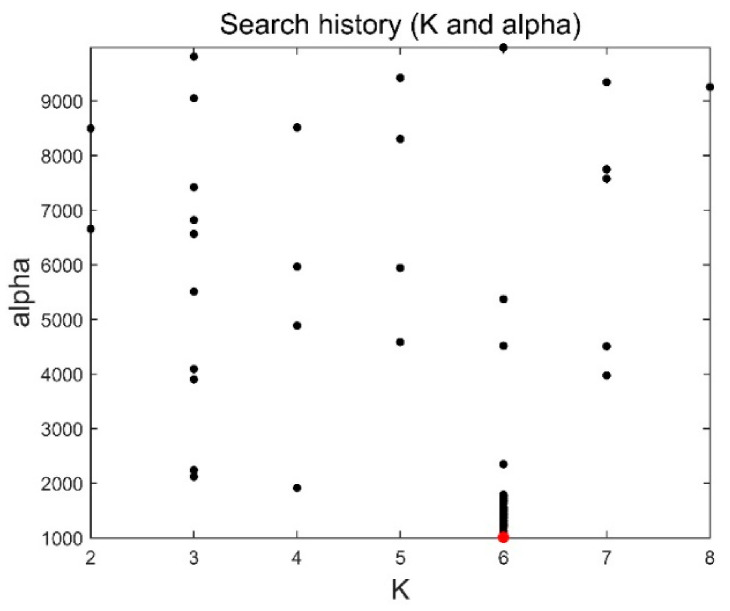
Historical values.

**Figure 5:**
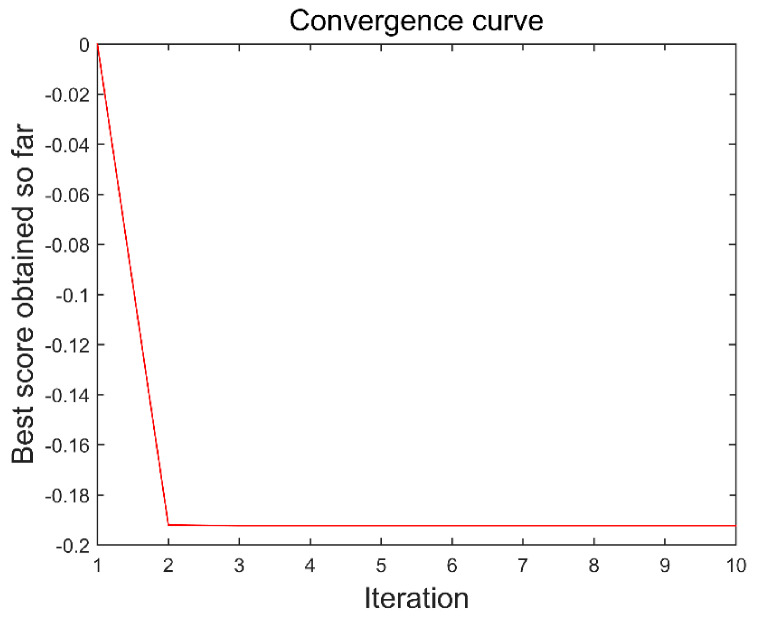
Convergence of the objective function of data I.

**Figure 6:**
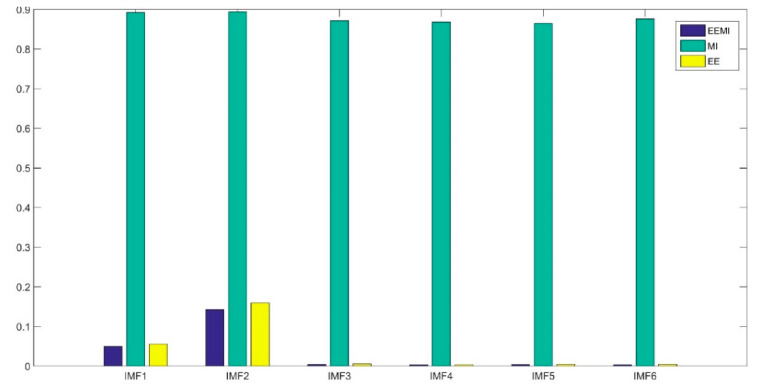
The three indexes for different IMFs.

The method in this paper was used to decompose simulated data A to select the optimal decomposition modal number K and penalty factor α of the VMD. The GOA parameters were set as follows: search agent n=30 and maximum number of loops L=10. Figure 4 and Figure 5 are the historical values of K and α and the convergent variation diagram of the objective function. Figure 4 and Figure 5 show that the optimal parameter combination of VMD is K=6 and α=1009. We prove the EEMI effectiveness by analysing the multiple indexes of the modes, including MI, EE and EEMI. As shown in Figure 6, the change in indexes MI was very small for the difference IMFs, while the indexes EE and EEMI obtained by GOA were larger than MI. It was indicated that EE and EEMI were sensitive to the two parameters of VMD, and the EEMI index was effective.

**Figure 7:**
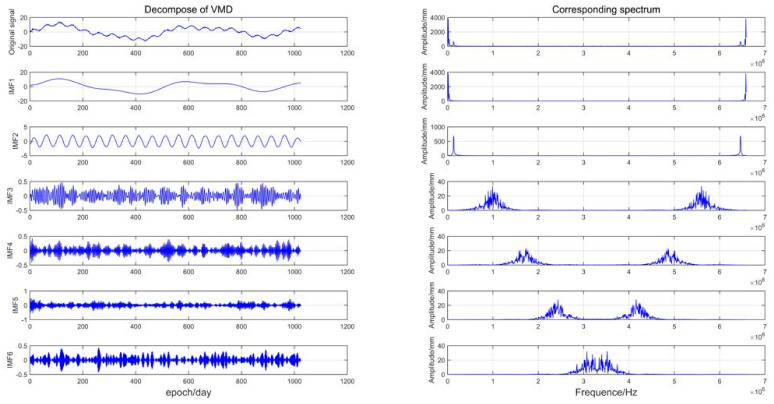
VMD decomposition and spectrum diagram.

The VMD method with the optimal parameter combination was used to decompose simulated data A. Figure 6 shows the decomposition result graph and the corresponding spectrum graph. Analysis of the spectrum diagram in Figure 7 shows that the IVMD decomposed the simulated data I into six IMFs, among which the IMF1–IMF3 modal component extracted the main information in the simulated data, while IMF4–IMF6 contained noise and some useful information. Then, to better distinguish high-frequency noise from low-frequency useful signals, the composite evaluation index T was adopted, and each modal component (IMF1–IMF6) was successively accumulated to form reconstructed time series, and the composite evaluation index T value of each reconstructed time series was calculated. The corresponding reconstructed time series was a denoising signal when the T value was minimal, and the remaining IMF components were regarded as high-frequency noise. Table 1 shows the corresponding T value of each reconstructed time series. When the reconstructed time series is $\sum_{i=1}^{2}IMF_{i}$, T=0.1697 reached the minimum, so $\sum_{i=1}^{2}IMF_{i}$ was regarded as a denoising time series and $IMF_{3}-IMF_{6}$ was a high-frequency noise.

As the high-frequency noise $IMF_{3}-IMF_{6}$ also contained part of the original time series information, wavelet packet analysis was carried out for further noise reduction. The parameters of wavelet packet: thresholding rule = 4.2975, thresholding function was hard, decomposition level number = 4, wavelet function = db3. The autocorrelation coefficient threshold method combined with EMD and IVMD was used to denoise the analogue time series, and a comparative analysis was performed with the method in this paper. The effect of denoising was evaluated using three indicators: root mean square error, correlation coefficient, and signal-to-noise ratio. The statistical table of the analysis results is shown in Table 2.

Table 2 shows that this method is superior to the EMD and IVMD methods on the whole, and the root mean square error of the simulated noise reduction results was 0.1563 mm. Compared with the EMD and IVMD methods, the root mean square error decreased by 0.084 and 0.0715, respectively, and the correlation coefficient was 0.9997. Compared with EMD and IVMD, the improvement was 0.0005 and 0.0004, respectively, thus verifying the effectiveness of the method.

### 4.2. Simulation Experiment B

**Figure 8:**
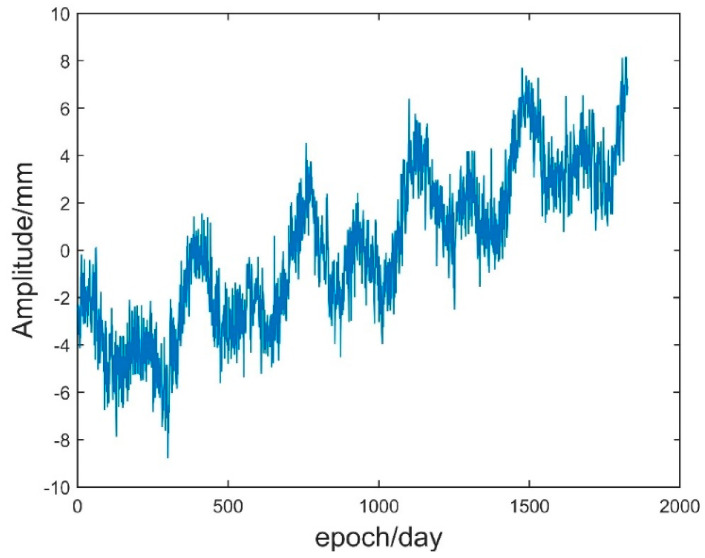
Simulated time series data.

The method in this paper was used to conduct noise reduction analysis on elevation data of GNSS coordinate time series. A simulated time series containing a trend term, periodic term and noise term was constructed according to the GNSS single station and single component coordinate time series function model and random model, and the function expression is as follows:

(28) $$y(t_{i})=a+\frac{bt_{i}}{T}+c\sin(\frac{2\pi t_{i}}{T})+d\cos(\frac{2\pi t_{i}}{T})+e\sin\frac{(4\pi t_{i}}{T})+f\cos(\frac{4\pi t_{i}}{T})+v_{i}$$

where $t_{i}$ is the observation time in units of year; a is the starting position of the time series of the station; b is the linear speed of the station movement; c,d,e,f are the amplitudes of the annual and semi-annual movements of the station, respectively; $v_{i}$ is the noise in the GPS coordinate time series, which is more realistic and simulates GPS time series noise. In this paper, Equation (28) was used to generate white noise and coloured noise to realistically simulate GPS time series noise.

(29) $$\left\{\begin{array}{c} v(t_{i})=w(t_{i}),i\leq 2\\ v(t_{i})=f_{1}\cdot v(t_{i-1})+f_{2}\cdot v(t_{i-2})+w(t_{i}),i\geq 3 \end{array}\right.$$

where $w(t_{i})$ is white noise with zero mean and variance of 1; $f_{1}$ and $f_{2}$ are 0.2 and 0.2, respectively. According to Equations (28) and (29), this paper generated simulated time series data II with a length of 1826. Table 3 shows the parameters of the simulated data, and Figure 8 shows the simulated time series.

**Figure 9:**
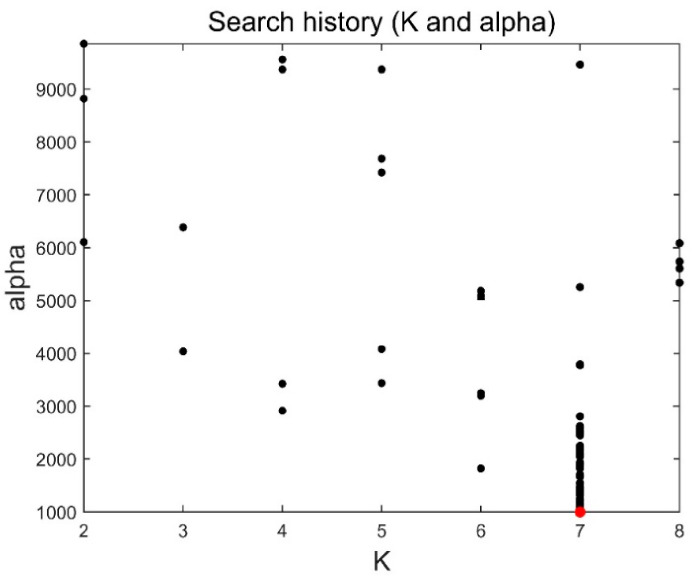
Historical values of simulated data II.

**Figure 10:**
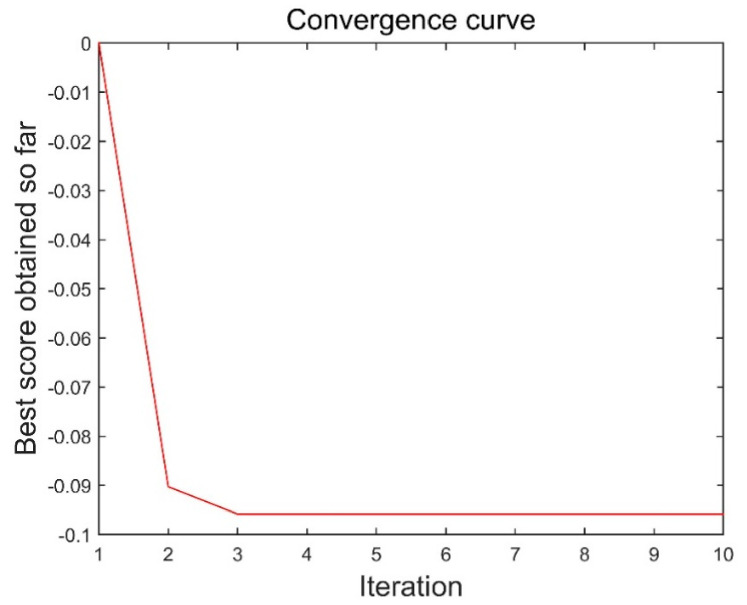
Convergence of the objective function of data Ⅱ.

According to the steps in Section 2.1, the EMD, IVMD, and IVMD-WPT methods were used to analyse the noise reduction of analogue data II. Figure 9 and Figure 10 show the parameter changes determined by the IVMD method, and Table 4 shows the corresponding T values of reconstructed time series. The analysis of Figure 8 and Figure 9 shows that the optimal parameter combination of VMD of the simulated data II was K=7 and α=1000. Combined with Table 4, the T index reached the minimum when the three IMF modal components were accumulated, so $\sum_{i=1}^{3}IMF_{i}$ will be used as the signal after denoising. Then, the root mean square error, correlation coefficient, and signal-to-noise ratio (SNR) after noise reduction using the EMD, IVMD and IVMD-WPT methods were calculated. Table 5 shows the statistical results of the three indicators.

By analysing the results in Table 5, it can be seen that the root mean square error decreased by 0.0087, and the correlation coefficient and the signal-to-noise ratio increased by 0.0007 and 0.7391, respectively. Therefore, for the GNSS coordinate time series data, the improved VMD method in this paper also had a better noise reduction effect than the EMD method. In the comparison between the IVMD-WPT method and IVMD method, the root mean square error decreased by 0.0003 and the signal-to-noise ratio increased by 0.0239, indicating that the IMF modal component that was removed also contained information. Therefore, the IVMD-WPT method proposed in this paper can extract effective information in the original time series more effectively.

### 4.3. Noise Reduction Analysis with Real GNSS Elevation Time Series

**Figure 11:**
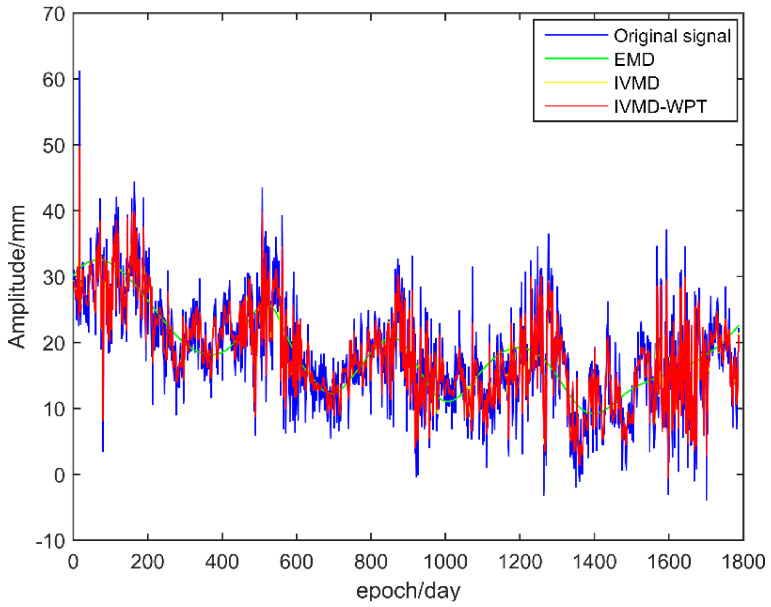
Noise reduction effect of the three methods for the BJFS station.

To further verify the reliability and applicability of the proposed method, this paper adopted the time series of the original elevation coordinates of 100 land state network reference stations in China to conduct noise reduction research (data came from the GNSS Data Product and Service Platform of the China Earthquake Administration). The observation epoch was a total of 5 years of elevation coordinate time series signals from 1 January 2010 to 1 January 2015, with a sampling interval of 1/365.25 a and a sampling frequency of 365.25 Hz. EMD, IVMD and the method in this paper were used to denoise the elevation data of 100 reference stations, and the relevant parameter settings were consistent with the simulation experiment. The BJFS station was taken as an example for detailed illustration. Figure 11 is the denoising effect diagram of the three methods for the BJFS station, and Table 6 is the statistical table of the denoising evaluation parameters of some stations.

As seen from Figure 10, compared with the EMD method, the IVMD-WPT method and IVMD method in this paper can better avoid the influence caused by the endpoint effect and have a better noise reduction effect at both ends of the original time series. The time series denoised by the IVMD-WPT method in this paper can better fit the original time series. It can effectively reflect local trend motion changes and small periodic oscillations. Table 3 summarises the root mean square error, signal-to-noise ratio and correlation coefficient of the results of the three algorithms. It can be seen from the table that the IVMD-WPT method in this paper is superior to the other two single methods on the whole. Compared with the other two methods, the IVMD-WPT method increased the root mean square error by 11.4648 and 6.7322 mm on average, respectively. The SNR index increased by 32.6773 and 26.3918 dB, and the correlation coefficient increased by 0.1458 and 0.0588, respectively, indicating that the noise reduction effect of the IVMD-WPT method presented in this paper was better and that the noise reduction results were more reliable.

## 5. Conclusions

Since the traditional VMD method cannot be sure of the number of decomposition mode functions (IMFs) and the value of the punishment factor in the process of noise reduction, resulting in inadequate decomposition or making the decomposition part a problem of denoising the time series effectively, a kind of energy entropy mutual information was proposed as the objective function to improve variational mode decomposition (VMD) combined with the wavelet packet denoising algorithm. The method was based on the traditional method of VMD, energy entropy mutual information as the objective function and the locusts optimised algorithm (GOA) to optimise the objective function; thus, the mode decomposition number and value of the punishment factor were determined adaptively, and the composite index T was used to determine the noise. Subsequently, the noise component was filtered using wavelet packet transform, and the filtered time series and denoising time series were reconstructed to obtain the final denoising time series. In this paper, two groups of analogue time series and 100 groups of measured time series of the land state network were used for research and analysis.

The main contributions of this approach are summarised below.

1. Compared with the traditional VMD method, this paper used the energy entropy mutual information as the objective function and used GOA to optimise the objective function to adaptively determine the number of decomposition mode functions (IMFs) and the value of the punishment factor and to improve the effect of noise reduction.
2. Compared with the single EMD method, the IVMD method can effectively weaken the influence of the endpoint effect, thus improving the noise reduction effect. The simulation results showed that the RMSE decreased by 0.0106 mm and the CC and SNR increased by 0.0004, and 428.42 dB, respectively.
3. Compared with the two single models of traditional EMD and IVMD proposed in this paper, the IVMD-WPT method was superior to the two single models in the three indicators of root mean square error, correlation coefficient and signal-to-noise ratio. The real results showed that the RMSE decreased by 11.4648 and 6.7322 mm and CC and SNR increased by 0.1458 and 0.0588 and 32.6773 and 26.3918 dB, respectively, thus verifying the effectiveness of the IVMD-WPT method in noise reduction. In addition, the local optimum problem of GOA (the appropriate parameters of WPT) need further exploration.

## Figures and Tables

**Table 1:** T indicators corresponding to different ${\mathrm{IMF}}_{s}$ of data I.

Reconstructed Time Series						
Index	$\sum_{i=1}^{1}IMF_{i}$	$\sum_{i=1}^{2}IMF_{i}$	$\sum_{i=1}^{3}IMF_{i}$	$\sum_{i=1}^{4}IMF_{i}$	$\sum_{i=1}^{5}IMF_{i}$	$\sum_{i=1}^{6}IMF_{i}$
T	0.6015	0.1679	0.1731	0.2089	0.2961	0.3985

**Table 2:** Statistical table of the evaluation parameters for different noise reduction methods in data Ⅰ.

Methods	RMSE/mm	Correlation Coefficient (R)	Signal-to-Noise Ratio (Rsn/dB)
EMD	0.2403	0.9992	637.95
IVMD	0.2278	0.9993	1494.06
IVMD-WPT	0.1563	0.9997	1500.23

**Table 3:** Simulation data statistics.

Time	Intercept (a)/mm	Linear Velocity (b)/(mm/a)	Annual Amplitude (c)/mm	Annual Amplitude (d)/mm	Half-Year Amplitude (e)/mm	Half-Year Amplitude (f)/mm	Period (T)
2013–2017	1	2	1	1.2	1.2	0.8	200

**Table 4:** T indicators corresponding to different ${\mathrm{IMF}}_{s}$ of data II.

Index	Reconstructed Time Series						
	$\sum_{i=1}^{1}IMF_{i}$	$\sum_{i=1}^{2}IMF_{i}$	$\sum_{i=1}^{3}IMF_{i}$	$\sum_{i=1}^{4}IMF_{i}$	$\sum_{i=1}^{5}IMF_{i}$	$\sum_{i=1}^{6}IMF_{i}$	$\sum_{i=1}^{7}IMF_{i}$
T	0.3616	0.3105	0.2931	0.3150	0.3773	0.4950	0.6384

**Table 5:** Statistical table of the evaluation parameters for different noise reduction methods in data II.

Methods	RMSE/mm	Correlation Coefficient (R)	Signal-to-Noise Ratio (Rsn/dB)
EMD	0.6546	0.9785	23.5371
IVMD	0.6459	0.9792	24.2762
IVMD-WPT	0.6456	0.9792	24.3001

**Table 6:** Statistical table of the noise reduction evaluation parameters.

Site	Methods	RMSE/mm	Correlation Coefficient (R)	Signal-to-Noise Ratio (Rsn/dB)
ARTU	EMD	4.8749	5.3318	0.9187
	IVMD	3.5984	10.3401	0.9568
	IVMD-WPT	2.5846	20.8624	0.9781
BJFS	EMD	8.8094	25.4094	0.9808
	IVMD	6.9204	41.3146	0.9882
	IVMD-WPT	3.2106	194.6615	0.9975
CHAN	EMD	5.8779	10.8899	0.9550
	IVMD	3.9240	23.9965	0.9800
	IVMD-WPT	2.5389	58.2924	0.9917
CHUN	EMD	5.3458	2.0200	0.8184
	IVMD	3.6352	5.0499	0.9215
	IVMD-WPT	2.9589	7.9185	0.9496
DLHA	EMD	142.7200	0.3229	0.0596
	IVMD	101.3457	0.2601	0.5831
	IVMD-WPT	36.8291	6.4418	0.9772
HRBN	EMD	5.7921	23.4629	0.9792
	IVMD	3.9684	50.0957	0.9903
	IVMD-WPT	2.8025	101.1486	0.9952
KMIN	EMD	7.5211	1.6570	0.7910
	IVMD	5.3586	3.7707	0.9017
	IVMD-WPT	3.1839	11.9409	0.9687
LUZH	EMD	4.0890	4.8302	0.9092
	IVMD	3.8465	5.2619	0.9203
	IVMD-WPT	2.4760	13.6532	0.9684
PIMO	EMD	4.5288	29.0476	0.9831
	IVMD	4.5896	27.6731	0.9827
	IVMD-WPT	4.1185	34.4699	0.9861
TAIN	EMD	5.2684	5.0086	0.9108
	IVMD	3.8686	9.3159	0.9533
	IVMD-WPT	2.7201	19.5294	0.9776
WUSH	EMD	5.1815	10.8638	0.9562
	IVMD	4.3153	15.2726	0.9699
	IVMD-WPT	2.9788	32.7606	0.9860
XIAG	EMD	6.6184	1.3874	0.7279
	IVMD	4.4656	3.3062	0.8854
	IVMD-WPT	2.6475	10.6794	0.9629

## Data Availability

Not applicable.

## Abbreviations

GPS	Global Positioning System
VMD	Variational Mode Decomposition
IMFs	Intrinsic Modal Functions
IVMD-WPT	Improved Variational Mode Decomposition and Wavelet Packet Transform
EEMI	Energy Entropy Mutual Information
GOA	Grasshopper Optimisation Algorithm
RMSE	Root Mean Square Error
CC	Correlation Coefficient
SNR	Signal-to-Noise Ratio
EMD	Empirical Mode Decomposition
IVMD	Improved Variational Mode Decomposition
CMONOC	Crustal Movement Observation Network of China
IGS	International GNSS Service
GNSS	Global Navigation Satellite System
LSWA	Least-Squares Wavelet Analysis
HHT	Hilbert–Huang Transform
EEMD	Ensemble Empirical Mode Decomposition
CEEMD	Complementary Ensemble Empirical Mode Decomposition

## References

[1] Bock Y., Melgar D. (2016). Physical applications of GPS geodesy: A review. Rep. Prog. Phys..

[2] He X., Montillet J.P., Fernandes R., Bos M., Yu K., Hua X., Jiang W. (2017). Review of current GPS methodologies for producing accurate time series and their error sources. J. Geodyn..

[3] Serpelloni E., Faccenna C., Spada G., Dong D., Williams S.D. (2013). Vertical GPS ground motion rates in the Euro-Mediterranean region: New evidence of velocity gradients at different spatial scales along the Nubia-Eurasia plate boundary. J. Geophys. Res. Solid Earth.

[4] Van Dam T.M., Blewitt G., Heflin M.B. (1994). Atmospheric pressure loading effects on the global positioning system coordinate determinations. Geophys. Res..

[5] Tregoning P., Watson C. (2009). Atmospheric effects and spurious signals in GPS analyses. Geophys. Res..

[6] Soundy A.W., Panckhurst B.J., Brown P., Martin A., Molteno T.C., Schumayer D. (2020). Comparison of Enhanced Noise Model Performance Based on Analysis of Civilian GPS Data. Sensors.

[7] Shen N., Chen L., Liu J., Wang L., Tao T., Wu D., Chen R. (2019). A Review of Global Navigation Satellite System (GNSS)-based Dynamic Monitoring Technologies for Structural Health Monitoring. Remote Sens..

[8] Han M., Liu Y., Xi J., Guo W. (2007). Noise smoothing for nonlinear time series using wavelet soft threshold. IEEE Signal Process Let..

[9] Altamimi Z., Rebischung P., Métivier L., Collilieux X. (2016). ITRF2014: A new release of the International Terrestrial Reference Frame modelling nonlinear station motions. J. Geophys. Res. Solid Earth.

[10] Ghaderpour E., Pagiatakis S.D. (2019). LSWAVE: A MATLAB software for the least-squares wavelet and cross-wavelet analyses. GPS Solut..

[11] Kaczmarek A., Kontny B. (2018). Identification of the noise model in the time series of GNSS stations coordinates using wavelet analysis. Remote Sens..

[12] Ghaderpour E., Pagiatakis S.D. (2017). Least-squares wavelet analysis of unequally spaced and non-stationary time series and its applications. Math Geosci..

[13] Huang N.E., Wu M.L., Qu W., Long S.R., Shen S.S. (2003). Applications of Hilbert–Huang transform to non-stationary financial time series analysis. Appl. Stoch. Models Bus..

[14] Baohong F., Yiqiang G. (2016). Application of Kalman filter in GPS data preprocessing. Sci. Surv. Mapp..

[15] Mosavi M.R., Rezaei M.J., Pashaian M., Moghaddasi M.S. (2017). A fast and accurate anti-jamming system based on wavelet packet transform for GPS receivers. Gps. Solut..

[16] Sun Z., Chang C.C. (2002). Structural damage assessment based on wavelet packet transform. J. Struct. Eng..

[17] Cody M.A. (1994). The wavelet packet transform: Extending the wavelet transform. DR Dobbs. J..

[18] Barros J., Diego R.I. (2007). Analysis of harmonics in power systems using the wavelet-packet transform. IEEE T Instrum. Meas..

[19] Huang N.E., Shen Z., Long S.R., Wu M.C., Shih H.H., Zheng Q., Yen N.C., Tung C.C., Liu H.H. (1971). The empirical mode decomposition and the Hilbert spectrum for nonlinear and non-stationary time series analysis. Proc. R. Soc. Math. Phys. Eng. Sci..

[20] Wang T., Zhang M., Yu Q., Zhang H. (2012). Comparing the application of EMD and EEMD on time-frequency analysis of seimic signal. J. Appl. Geophys..

[21] Liu C., Zhu L., Ni C. (2017). The chatter identification in end milling based on combining EMD and WPD. Int. J. Adv. Manuf. Technol..

[22] Zheng J.D., Cheng J.S., Yang Y. (2013). Modified EEMD algorithm and its applications. J. Vib..

[23] Shen W., Peng C. (2016). Detection of different-time-scale signals in the length of day variation based on EEMD analysis technique. Geod. Geodyn..

[24] Wang J., He X., Ferreira V.G. (2015). Ocean wave separation using CEEMD-Wavelet in GPS wave measurement. Sensors.

[25] Lu J., Chen X., Feng S. (2016). A GPS Time Series Prediction Model Based on CEEMD. J. Adv. Comput. Netw..

[26] Zhang J., Tang H., Wen T., Ma J., Tan Q., Xia D., Zhang Y. (2020). A hybrid landslide displacement prediction method based on CEEMD and DTW-ACO-SVR—Cases studied in the three gorges reservoir area. Sensors.

[27] Zhang J., Tang H., Tannant D.D., Lin C., Xia D., Liu X., Ma J. (2021). Combined forecasting model with CEEMD-LCSS reconstruction and the ABC-SVR method for landslide displacement prediction. J. Clean. Prod..

[28] He X., Yu K., Montillet J.P., Xiong C., Lu T., Zhou S., Ma X., Cui H., Ming F. (2020). GNSS-TS-NRS: An Open-source MATLAB-Based GNSS time series noise reduction software. Remote Sens..

[29] Zhang S., Liu H., Hu M., Jiang A., Zhang L., Xu F., Hao G. (2020). An adaptive CEEMDAN thresholding denoising method optimized by nonlocal means algorithm. IEEE Trans. Instrum. Meas..

[30] Dragomiretskiy K., Zosso D. (2014). Variational Mode Decomposition. IEEE Trans. Signal Process..

[31] Shen Y., Zheng W., Yin W., Xu A., Zhu H. (2021). Feature Extraction Algorithm Using a Correlation Coefficient Combined with the VMD and Its Application to the GPS and GRACE. IEEE Access.

[32] Zhang X., Miao Q., Zhang H., Wang L. (2018). A parameter-adaptive VMD method based on grasshopper optimization algorithm to analyze vibration signals from rotating machinery. Mech. Syst. Signal PR.

[33] Ahmed W.A., Wu F., Marlia D., Zhao Y. (2019). Mitigation of Ionospheric Scintillation Effects on GNSS Signals with VMD-MFDFA. Remote Sens..

[34] Zhou C., Ma J., Wu J., Yuan X. (2019). An adaptive VMD method based on improved GOA to extract early fault feature of rolling bearings. Int. J. Innov. Comput. Inf. Control.

[35] Li C., Liu Y., Liao Y. (2021). An Improved Parameter-Adaptive Variational Mode Decomposition Method and Its Application in Fault Diagnosis of Rolling Bearings. Shock Vib..

[36] Saremi S., Mirjalili S., Lewis A. (2017). Grasshopper optimisation algorithm: Theory and application. Adv. Eng. Softw..

[37] Meng H., Han Y., Xun Y., Chen J., Du N., Cao Y., Zheng Y. (2019). GPS/INS Integrated Navigation Based on Grasshopper Optimization Algorithm. IFAC-Papers Online.

[38] Mafarja M., Aljarah I., Heidari A.A., Hammouri A.I., Faris H., Ala’M A.Z., Mirjalili S. (2018). Evolutionary population dynamics and grasshopper optimization approaches for feature selection problems. Knowl. Based Syst..

[39] Jiang X., Shen C., Shi J., Zhu Z. (2018). Initial center frequency-guided VMD for fault diagnosis of rotating machines. J. Sound Vib..

[40] He X., Bos M.S., Montillet J.P., Fernandes R., Melbourne T., Jiang W., Li W. (2021). Spatial Variations of Stochastic Noise Properties in GPS Time Series. Remote Sens..

[41] Silva L.K.J., Ramarakula M. (2021). An efficient interference mitigation approach for NavIC receivers using improved variational mode decomposition and wavelet packet decomposition. Trans. Emerg. Telecommun. Technol..

[42] Zhu J., Zhang Z., Kuang C. (2015). A Reliable Evaluation Indicator of Wavelet Denoising. Geomat. Inf. Sci. Wuhan Univ..

